# Macrosurgery for fingertip amputations

**DOI:** 10.1186/1753-6561-9-S3-A58

**Published:** 2015-05-19

**Authors:** Marcos Sanmartin Fernandez

**Affiliations:** 1 Department of Orthopaedics and Traumatology, Hospital Povisa, Vigo, 36211, Spain

## 

Due to the high concentration of sensitive receptors, the fingertip is widely used in plenty of activities and, therefore, frequently exposed to trauma and amputation. Simple revision amputation was used in the past for the treatment of these injuries. However, replantation, revascularization, or reconstruction allows preservation of the nail, length of the digit, and, mainly, sensibility of the pulp.

Undoubtedly, the best way to restore function to an amputated digit is to restore the amputated part by microsurgical vascular anastomosis. However, the replantation of very distal segments is technically challenging due to the size and fragility of the vessels. Furthermore, the amputated part is not always available.

Reattachment of the amputated part without vascular anastomosis is described as composite graft [1]. Other authors described different ways for improving the success rate. Brent [2] described the reattachment in amputations that occurred between the lunula and the DIP joint by performing deepithelization of the amputated part and embedding it in a subcutaneous pocket in the contralateral chest. Arata et al. [3] modified this procedure using the ipsilateral palm as the pocket site.

We consider subzone 1 Ishikawa level as suitable for reconstruction without replantation. In this case, a local flap can adequately restore function and appearance. The neurovascular homodigital advancement flap [4] is the most useful flap for coverage of the amputated part. For larger areas that cannot be covered with a homodigital advancement flap, we use innervated cross finger flaps [5].

The Moberg flap is a good option to reconstruct medium-size defects of the pulp of the thumb [6]. For covering larger areas, we use the first metacarpal island flap [7]. Raising a racket flap as proposed by Holevich [8] adds safety regarding venous congestion, once a subcutaneous tunnel is avoided, and can improve cold intolerance, since it transfers more nerve branches to the thumb allowing for a better temperature control [9].

The nail complex tissues, as hyponychium, paronychium, and sterile matrix cannot be reconstructed with local tissue. If the amputated part is available, these parts can be used as grafts applied to the flap used to reconstruct the pulp[10].

The eponychial flap [11] can be used to increase the exposed part of the nail bed. This is achieved by removing a rectangle of dorsal skin and performing a proximal translocation of the eponychium.
